# Phase II trial of single-agent foretinib (GSK1363089) in patients with recurrent or metastatic squamous cell carcinoma of the head and neck

**DOI:** 10.1007/s10637-012-9861-3

**Published:** 2012-08-24

**Authors:** Tanguy Seiwert, John Sarantopoulos, Howard Kallender, Stewart McCallum, Harold N. Keer, George Blumenschein

**Affiliations:** 1 The University of Chicago, 5841 S Maryland Ave, MC2115, Chicago, IL 60637 USA; 2 University of Texas Health Science Center, San Antonio, TX USA; 3 GlaxoSmithKline, Collegeville, PA USA; 4 Exelixis, South San Francisco, CA USA; 5 The University of Texas MD Anderson Cancer Center, Houston, TX USA

**Keywords:** Head and neck cancer, c-MET, VEGFR2, Foretinib, Phase II study

## Abstract

*Background* Foretinib is a small-molecule, oral multikinase inhibitor primarily targeting the mesenchymal epithelial transition (MET) factor receptor, and the vascular endothelial growth factor receptor 2. We conducted a phase II study to evaluate the single-agent activity and tolerability of foretinib in patients with recurrent/metastatic squamous cell carcinoma of the head and neck (SCCHN). *Methods* An open-label, single-arm, multicenter trial employing a Simon 2-stage design was conducted with a total of 41 patients planned for the study. One or more responses in the first 14 patients were required in order to progress to the second stage. Foretinib was administered as 240 mg orally for 5 consecutive days of a 14-day treatment cycle (5/9 schedule) to patients with recurrent and/or metastatic SCCHN. *Results* Fourteen patients were enrolled. The study did not meet criteria for continuing to the second stage. A maximum of 30 cycles were administered (median = 4.0). Fifty percent of patients (7/14) showed stable disease (SD), 43 % of patients (6/14) experienced tumor shrinkage and two patients had prolonged disease stabilization for ≥13 months. The most common adverse events were fatigue, constipation and hypertension, which were manageable with additional medication or adjustments to the dosing schedule. *Conclusion* Foretinib 240 mg on a 5/9 schedule was generally well tolerated. SD was the best-observed outcome, with minor tumor shrinkage detected in nearly half of all patients. The efficacy results, prolonged disease stabilization and tolerable side-effect profile, support further investigation, possibly in combination with other targeted agents or cytotoxic chemotherapy for SCCHN.

**Electronic supplementary material:**

The online version of this article (doi:10.1007/s10637-012-9861-3) contains supplementary material, which is available to authorized users.

## Introduction

Head and neck cancer (HNC) represents a heterogeneous group of potentially deadly cancers. Approximately 650,000 HNC diagnoses are made each year worldwide with almost 50,000 cases and 11,000 deaths occurring in the United States alone [1, 2]. Overall 5-year survival rates for patients with HNC are below 50 % [3]. Although the head and neck comprise a variety of tissue types, squamous cell carcinomas originating from mucosal surfaces represent >90 % of all cases of HNC [3]. The risk factors for squamous cell carcinomas of the head and neck (SCCHN) have been strongly linked with tobacco and alcohol use as well as with human papillomavirus [3].

Treatment modalities for SCCHN include surgery, radiation therapy, and chemotherapy [4]. Most patients with SCCHN present with advanced locoregional disease [5]. With advanced SCCHN, only 35 % to 55 % of patients survive and remain disease-free for 3 years, despite aggressive therapy [1]. Locoregional recurrence develops in 30 % to 40 % of patients and distant metastases develop in 12 % to 22 % of patients [6]. Once the disease is recurrent/metastatic, combination chemotherapy using a platinum-based regimen remains the standard of care for SCCHN. The addition of cetuximab, an epidermal growth factor receptor (EGFR) tyrosine kinase inhibitor, to chemotherapeutic agents has provided a modest survival benefit (10 vs. 7 months) [7]. Nevertheless, palliative treatment of recurrent/metastatic SCCHN remains largely ineffective and little progress has been made. More effective, targeted treatments are needed.

The mesenchymal epithelial transition (MET) factor receptor and its sole ligand, hepatocyte growth factor (HGF), are strongly overexpressed in >80 % of SCCHN [8, 9]. Activation of the MET factor receptor by HGF stimulates cell proliferation, survival and motility, thus promoting cancer cell metastases [9, 10]. There is evidence to suggest that the HGF/MET signaling pathway may represent a promising target in the treatment of recurrent SCCHN, as preclinical data have provided additional support for activity with MET inhibitors in SCCHN models [8, 9, 11]. Vascular endothelial growth factor (VEGF) is also upregulated in patients with SCCHN [12]. VEGF upregulation in SCCHN has been linked to tumor angiogenesis and disease severity [13, 14]. However, targeting VEGF receptors alone in recurrent or metastatic cancers of the head and neck has shown modest objective response rates; data from one study showed only one minor response and one partial response out of 31 evaluable patients [15]. HGF and VEGF may, therefore, act in concert to spur angiogenesis and metastasis in patients with SCCHN. Thus, targeting both the HGF/MET and VEFG signaling pathways is an attractive therapeutic approach [16]. Previous studies combining anti-angiogenic agents with other approaches have shown promise with combination approaches using anti-angiogenic agents, such as the combination of erlotinib, an EGFR tyrosine kinase inhibitor, and bevacizumab [17]. Other targeted therapies used as single agents have shown limited or no activity for SCCHN [18].

Foretinib (also known as GSK1363089 or XL880) is an oral multikinase inhibitor that primarily targets signaling of HGF/MET (*in vitro* IC_50_ of 3 nmol/L) and the VEGF receptor-2 (VEGFR2) (*in vitro* IC_50_ of 7 nmol/L) [19] signaling pathways by binding in the adenosine triphosphate pocket of both MET and VEGFR2. In preclinical studies, foretinib induced tumor hemorrhage and necrosis in human xenografts [20]. Foretinib also targets several other receptor tyrosine kinases (RTKs), including the MET-related recepteur d'origine nantais (RON) receptor (*in vitro* IC_50_ of 3 nmol/L) [19] and additional RTKs involved in tumor angiogenesis (AXL and TIE-2) [20, 21]. While the role of RON in SCCHN remains unclear, its effects appear to largely overlap with MET, and in other tumor types synergy has been reported with the inhibition of both targets [22]. MET has been evaluated extensively as a potential treatment target for SCCHN, with promising results [8, 9], and foretinib demonstrated activity against human SCCHN cell lines [23]. Foretinib has also shown antitumor activity in clinical studies of papillary renal cell carcinoma and hepatocellular carcinoma [24, 25], and may have the potential to prevent tumor growth in SCCHN, chiefly by reducing tumor cell proliferation and metastasis through HGF/MET inhibition and decreasing angiogenesis through VEGFR2 pathway inhibition.

The primary purpose of this study was to evaluate the response rate for single-agent foretinib treatment in patients with recurrent and/or metastatic SCCHN, and to assess foretinib safety and tolerability in SCCHN patients. This is the first report evaluating a MET inhibitor in SCCHN.

## Patients and methods

Eligible patients were ≥18 years of age with histologically or cytologically confirmed recurrent and/or metastatic SCCHN who were not eligible for curative-intent surgery or radiotherapy. Patients had measurable disease according to Response Evaluation Criteria In Solid Tumors (RECIST) 1.0, Eastern Cooperative Oncology Group (ECOG) performance status of ≤1 [26]. All patients participating in the study provided informed consent.

Exclusion criteria included previous radiation therapy (>25 % of bone marrow) within 30 days of study treatment, >1 regimen of systemic anticancer therapy for disease that had recurred or was metastatic, except for adjuvant or neoadjuvant chemotherapy, those who had disease progression within 6 months after completion of curative-intent therapy, and patients at high risk of bleeding.

### Study design

This was a single-arm, phase II, multicenter (all in the United States), non-randomized, open-label, Simon 2-stage safety and efficacy study [27]. The primary objectives of this study were to determine the response rate according to RECIST 1.0 [28] for foretinib treatment in patients with SCCHN, and to evaluate the safety and tolerability of foretinib. Secondary objectives included an assessment of progression-free survival (PFS), duration of response, overall survival (OS) and the pharmacokinetic parameters of foretinib. Foretinib was administered at doses of 240 mg orally for 5 consecutive days of a 14-day treatment cycle (5/9 schedule). Patients fasted from 2 h prior to 1 h after each dose. In the absence of progressive disease and unacceptable toxicity, patients were eligible to continue with foretinib treatment for 1 year or longer. If the patient required additional anticancer therapy (e.g. chemotherapy, radiation or surgery), foretinib dosing was discontinued. The relationship between foretinib trough concentrations and percent change from baseline in tumor size was examined.

### Assessments

Tumor assessments were performed within 14 days before dosing. During the study treatment period tumor response was assessed after 8 weeks. Patients were asked to return to the study site 30 days after the last dose of foretinib for laboratory assessments and clinical examination. Patients were contacted for follow-up at 90 and 180 days after the last dose. Toxicity grade of adverse events (AEs), serious AEs and laboratory variables were defined by the National Cancer Institute Common Terminology Criteria for Adverse Events v3.0.

### Statistical analyses

A total of 14 patients were enrolled into stage 1 (to ensure a total of 12 evaluable patients). If no patients had either a complete response (CR) or partial response (PR) to treatment in stage 1, then the study was to be halted. If one or more patients had a response in stage 1, a second stage was to be opened to enroll additional patients up to a total of 41 patients (to ensure a total of 35 evaluable patients). EGFR inhibitors have a response rate of 5 % to 13 % as single agents, and a similar response rate was hypothesized to be meaningful in the current study [7, 29]. The study had a type 1 error rate of 5 % for the null hypothesis that the response rate is at least 10 %, with 80 % power for an alternative response rate of at least 25 %.

Response rates were summarized with exact 95 % confidence intervals using Klopper–Pearson methods, and PFS, stable disease (SD) and OS data were summarized using Kaplan–Meier methods with 95 % confidence intervals for medians. Foretinib exposure measures for all analyses were the average trough concentration for foretinib across days 5, 19, 33 and 47, which represented the trough concentration after 4 days of dosing. Ordinal logistic regression was used to examine the maximal grade of the following AEs: elevated aspartate aminotransferase (AST), elevated alanine aminotransferase (ALT), elevated lactate dehydrogenase, fatigue and hypertension. Linear regression analysis was used to determine whether a relationship existed between exposure and change in tumor size.

## Results

### Patient disposition

Between August 2007 and May 2009, 14 patients were enrolled. Of these, only 11 treated patients had at least one on-treatment scan. Recruitment was halted because no patient met the treatment response criterion (CR or PR) required for continuation to stage 2. All patients had histologically or cytologically confirmed SCCHN, with a mean time since the initial diagnosis of 1.4 years (range, 0–7 years). All patients had distant metastatic disease, and 12 patients had received prior antitumor therapy regimens and radiation therapy (85.7 %). Two patients did not have any line of prior therapy for recurrent and/or metastatic disease, four patients had received only first-line therapy, six patients two lines of prior therapy, and two patients three lines of prior therapy. Chemotherapeutic drugs included platinating agents, taxanes, 5-FU, hydroxycarbamide, cetuximab and bevacizumab. Table 1 presents the baseline demographics and Table 2 displays the disposition of the 14 patients included in the study.

**Table 1:** Baseline demographics

Category	Measure
Age, years, median (range)	59.0 (48–82)
Male, n (%)	13 (92.9)
Race, n (%)	
Asian	1 (7.1)
White	12 (85.7)
Other	1 (7.1)
ECOG performance status, n (%)	
0	9 (64.3)
1	5 (35.7)
Cancer history, n (%)	
Laryngeal	1 (7.1)
Oropharyngeal	4 (28.6)
Other	9 (64.3)
Initial cancer staging at diagnosis, n (%)	
II	2 (14.3)
III	1 (7.1)
IV	7 (50.0)
Unknown	4 (28.6)
Sites of metastases, n (%)	
Bone	1 (7.1)
Lymph node	10 (71.4)
Liver	3 (21.4)
Lung	12 (85.7)
Other	3 (21.4)

*ECOG* Eastern Cooperative Oncology Group

**Table 2:** Patient disposition

Reason for discontinuation	Patients, n (%)
Withdrawn by patient	2 (14.3)
Physician decision	1 (7.1)
Progressive disease	7 (50.0)
Lost to follow-up	1 (7.1)
Death	1 (7.1)
Other	2 (14.3)
Total discontinued	14 (100)

### Efficacy

**Fig. 1:**
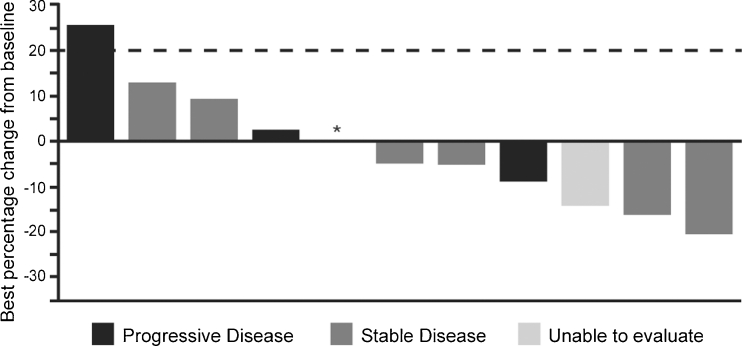
Waterfall plot for best percentage change from baseline in target lesion tumor measurement. Only 11 of the 14 treated patients had at least one on-treatment scan to be included. *Patient had a best percentage change from baseline in tumor measurement of 0 %. At that visit, the overall response assessment was stable disease

Although there were no confirmed PRs or CRs in this trial, seven of 14 patients had SD and six of 14 patients experienced some tumor shrinkage (range 5–21 %) (Fig. 1). The median duration of SD was 4.1 months and the disease stabilization rate was 50 % (Table 3). Two patients had prolonged SD of 13 and 13.9 months’ duration, respectively.

**Table 3:** Tumor response and survival outcomes

Best overall response, n (%)	
Complete response	0
Partial response	0
Stable disease	7 (50.0)
Disease progression	3 (21.4)
Unable to evaluate	1 (7.1)
Objective response rate, % (95 % CI)	0 (0–23.2)
Disease stabilization rate^a^, % (95 % CI)	7 (50) (23.0–77.0)
Duration of stable disease^b^, months (95 % CI)	4.11 (3.65–13.86)
Progression-free survival, months, median (95 % CI)	3.65 (3.4–5.3)
Overall survival, months, median (95 % CI)	5.59 (3.71–NA)

^a^Proportion of patients achieving a best overall response of complete response, partial response, or stable disease

^b^Only patients whose best overall response was not disease progression were included

*CI* confidence interval

**Fig. 2:**
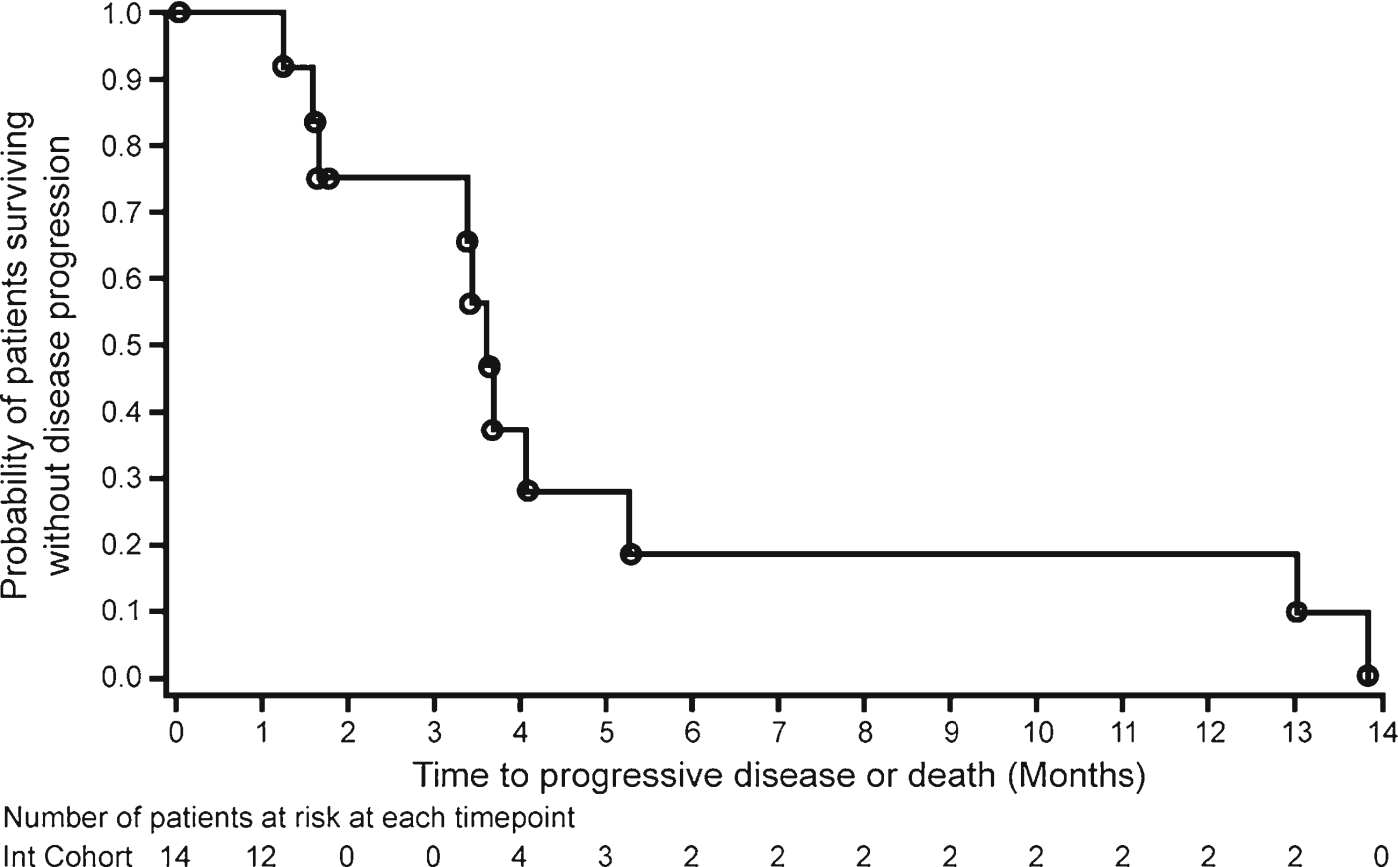
Kaplan–Meier progression-free survival curve (foretinib administered using intermittent dosing 5/9 schedule)

**Fig. 3:**
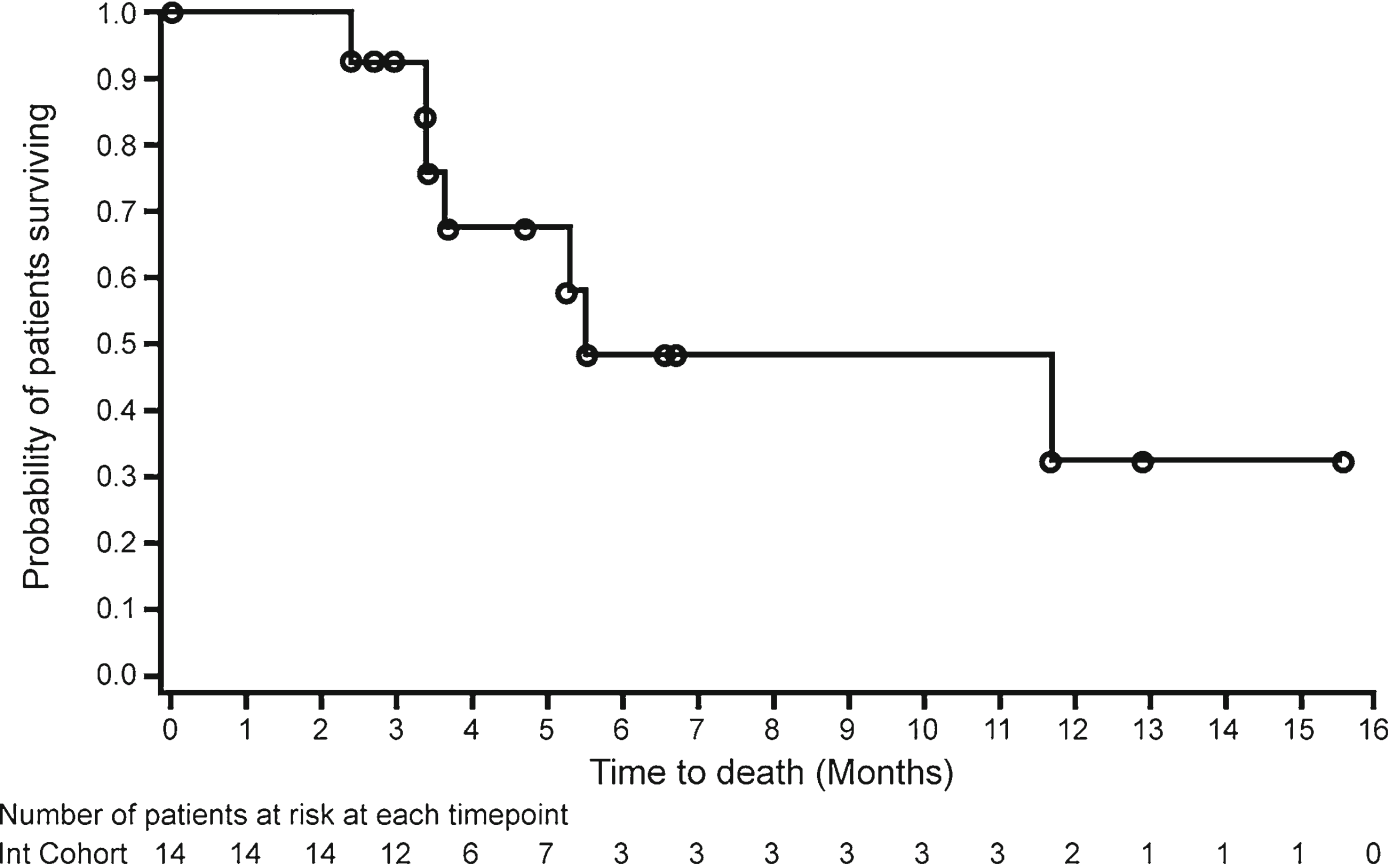
Kaplan–Meier overall survival curve (foretinib administered using intermittent dosing 5/9 schedule)

The median duration of PFS was 3.65 months (Fig. 2). The median OS was 5.59 months; five patients (35.7 %) were alive at 6 months and two patients (14.3 %) at 12 months (Fig. 3).

### Pharmacokinetics

For the exposure-response analysis, foretinib trough concentration data were available for 11 patients; however, week 8 tumor size data were available for only nine patients. No relationship was detected between average foretinib trough concentrations after 4 days of dosing and percent change from baseline to week 8 in the sum of the longest tumor diameter (Supplemental Fig. 1).

Moreover, no relationship was detected between average foretinib trough concentrations and the incidence of any AE. Statistical tests were limited by the small sample size.

### Safety and adverse events

All patients experienced at least one AE with 14 patients evaluable for safety. Fatigue, constipation and hypertension were the most common AEs, occurring in seven (50 %), five (35.7 %), and five (35.7 %) patients, respectively (Table 4). These were managed with additional medication (e.g. antihypertensives) or dose delay and/or reduction. Of all AEs, 55 % were considered related to foretinib treatment. The most frequent treatment-related grade 3 AE was hypophosphatemia (experienced by three patients). There were no grade 4 treatment-related AEs, but one fatal hemorrhage occurred during cycle 7 and was considered possibly related to foretinib. This patient had severe hemoptysis, and cardiopulmonary resuscitation was unsuccessful.

**Table 4:** Most common (>2 patients) adverse events

Event^a^	Patients, n (%)	Grade 3, 4, or 5, n (%)
Fatigue	7 (50.0)	2 (14.3)
Constipation	5 (35.7)	–
Hypertension	5 (35.7)	1 (7.1)
Alanine aminotransferase increase	4 (28.6)	–
Anorexia	4 (28.6)	–
Aspartate aminotransferase increase	4 (28.6)	–
Dysphagia	4 (28.6)	1 (7.1)
Dyspnea	4 (28.6)	1 (7.1)
Headache	4 (28.6)	–
Mucosal inflammation	4 (28.6)	–
Weight decrease	4 (28.6)	–
Dehydration	3 (21.4)	2 (14.4)
Depression	3 (21.4)	–
Dizziness	3 (21.4)	–
Dry skin	3 (21.4)	–
Hypophosphatemia	3 (21.4)	3 (21.4)
Insomnia	3 (21.4)	–
Musculoskeletal pain	3 (21.4)	–
Nausea	3 (21.4)	–
Rash	3 (21.4)	–

^a^Treatment-emergent AE was defined as any AE with an onset date on or after the date of first dose of study drug, or any ongoing event that worsened in intensity after the date of first dose, but before the date of last dose plus 30 days

*AEs* adverse events

A total of six other patients died during treatment or follow-up. Four (28.6 %) of these deaths were due to progressive disease and two (14.3 %) due to other causes (pneumonia/respiratory failure and reasons that were not confirmed); none of these six deaths were attributed to the study medication. Two of 14 patients (14 %) required dose reductions to 160 mg due to AEs. No AEs occurred that resulted in study drug discontinuation.

## Discussion

Recurrent and/or metastatic SCCHN is a devastating disease for which few effective treatment options are available. This is the first report evaluating a MET inhibitor as a single agent for SCCHN. While the response rate in this two-stage phase II trial did not meet the criteria to allow progression to stage 2, as there were no responders based on RECIST, signs of moderate activity were evident: seven of 14 patients (50 %) experienced SD and six of 14 patients (43 %) showed tumor shrinkage of up to 21 %. Two patients (2/14) (14 %) experienced SD over a period of 13 months or more, exceeding the typical PFS of 3–5 months observed with standard of care [7, 30].

Cetuximab is commonly used in the treatment of recurrent/metastatic disease, either in combination with chemotherapy or as a single agent [31, 32]. The addition of cetuximab to chemotherapy as first-line treatment of patients with recurrent or metastatic SCCHN increased the response rate from 20 % to 36 % (*P* < 0.001) and median PFS from 3.3 to 5.6 months (*P* < 0.001) [31]. As a single agent, cetuximab has demonstrated only moderate activity in a phase II study; the best response rate was 13 % (13/103 patients) and 33 % (34/103 patients) experienced SD [32]. The disease control rate was 46 % and the median TTP was 2.3 months [32]. Methotrexate as a single agent has been a standard comparator for clinical studies and has shown response rates of only 3.9 % (6/152 patients; PFS data not reported) [33]. By comparison, foretinib in this (albeit much smaller) study showed a PFS of 3.65 months and a disease stabilization rate of 50 %.

There was no biomarker analysis performed in this study to predict a response to treatment, and it is unclear from the information obtained why the tumors did not respond as predicted by the preclinical data. However, there are many factors that could play a role: foretinib levels in the tumor cells may not be high enough to sufficiently inhibit MET with the intermittent 5/9 schedule, although pharmacodynamic data and clinical data in papillary renal cell [24, 25] and hepatocellular carcinoma [34] do support adequate target inhibition [20]. Notably, inhibition of MET phosphorylation and decreased proliferation in selected tumor biopsies were observed in patients treated with submaximal doses of foretinib [20]. A more likely explanation may be that the *in vivo* situation is more complex than suggested by *in vitro* models. One hypothesis may be that additional pathways to those targeted by foretinib may contribute to MET resistance. In future studies, serial biopsy may help elucidate mechanisms of resistance.

We know from other cancer types that compensatory RTK signaling can lead to tumor robustness and resistance [22, 35], and co-targeting may increase efficacy. In non-small cell lung cancer (NSCLC), MET inhibition increased the efficacy of the EGFR inhibitor erlotinib in two large randomized phase II trials [11, 36], despite limited single-agent activity of the respective MET inhibitors in NSCLC [37]. It is therefore reasonable to hypothesize that combined MET/EGFR inhibition may be a promising approach for SCCHN. This is supported by preclinical data showing potent MET/EGFR synergy in SCCHN cell line models [11, 21].

While this is a negative study that did not meet the predefined statistical criteria to proceed to stage 2 of the trial, it is also evident that there is modest activity in a high proportion of SCCHN patients. If significant inhibition of invasion and metastasis is achieved by foretinib treatment, this may be clinically meaningful and efficacy assessment by RECIST may not be optimal in assessing true clinical benefit. In a recent study, time to development of new metastatic lesions was evaluated with a different MET inhibitor (tivantinib) in combination with erlotinib in patients with advanced NSCLC [38]. Time to new metastasis was delayed with tivantinib plus erlotinib versus erlotinib alone (7.3 vs 3.6 months, respectively; *P* < 0.01). The effect was more pronounced in patients with non-squamous histology (median time to metastatic disease 11.0 vs 3.6 months, respectively; *P* < 0.01), whilst the objective response rates were only 10 % (7/74 patients) vs 7 % (5/72 patients), respectively (PRs only) [38]. These data further support the evaluation of time to metastatic disease with MET inhibitors in follow-up studies. Furthermore, the anti-angiogenic effect of foretinib may result in a metabolic response against the tumor rather than tumor shrinkage. Future studies should take this into consideration as well as incorporating biomarker analyses to help understand and predict a response to treatment.

Foretinib was well tolerated, with the most common AEs (fatigue, constipation and hypertension) being readily manageable, and the most common foretinib-related laboratory abnormalities (elevated ALT and AST) being asymptomatic. Thus, foretinib may be a good candidate for combination therapy. At this point, no validated biomarkers are available and the small sample size precludes further analysis. There are several potential biomarker candidates, including MET immunohistochemistry [39] and MET copy number [38].

Foretinib is not a MET-specific inhibitor. Like many MET tyrosine kinase inhibitors, it also inhibits the MET-like kinase RON, which is functionally similar to MET [22]. In contrast to other more specific MET inhibitors, foretinib also inhibits VEGFR2 and TIE-2 at clinically achievable concentrations. Inhibition of these multiple targets may have contributed to the modest activity seen; however, further exploration is required [40]. The activity of foretinib against targets in addition to MET may provide a good basis for achieving better outcomes with combination therapy in the future.

No AEs related to MET inhibition were reported; however, AEs related to VEGF inhibition were observed. Observations included night blindness in some patients receiving foretinib [24], other ocular toxicities with crizotinib [41], a MET/ALK inhibitor, and hematologic toxicities with tivantinib [42], an inhibitor of MET. The fact that the three MET inhibitors do not result in similar toxicities suggests that these effects may be potentially unrelated to MET inhibition.

In conclusion, this is the first report of a MET inhibitor used for SCCHN. There is evidence of modest activity, despite the lack of objective responses to treatment. Furthermore, preclinical data, as well as clinical observations, in NSCLC suggest that combination approaches with EGFR inhibition may be promising and should be explored further.

## Electronic supplementary material

Below is the link to the electronic supplementary material.

ESM 1 Relationship between foretinib trough concentration and percentage change from baseline in tumor size. Average trough concentration on study days 5, 19, 33 and 47 (JPEG 10 kb)

High Resolution Image 1 (TIFF 756 kb)
