# Supplementary figures and images for: Phase II trial of single-agent foretinib (GSK1363089) in patients with recurrent or metastatic squamous cell carcinoma of the head and neck
Source: Invest New Drugs. 2012 Aug 24;31(2):417–24. doi: 10.1007/s10637-012-9861-3 (PMC3589657; doi:10.1007/s10637-012-9861-3)

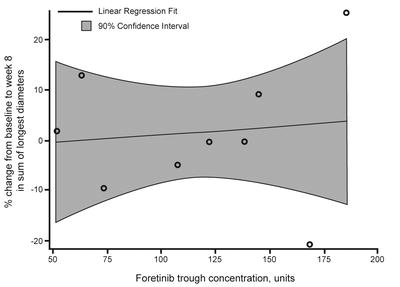

Supplement: Supplementary file 1 — Relationship between foretinib trough concentration and percentage change from baseline in tumor size. Average trough concentration on study days 5, 19, 33 and 47 (JPEG 10 kb) [file 10637_2012_9861_Fig4_ESM.jpg]

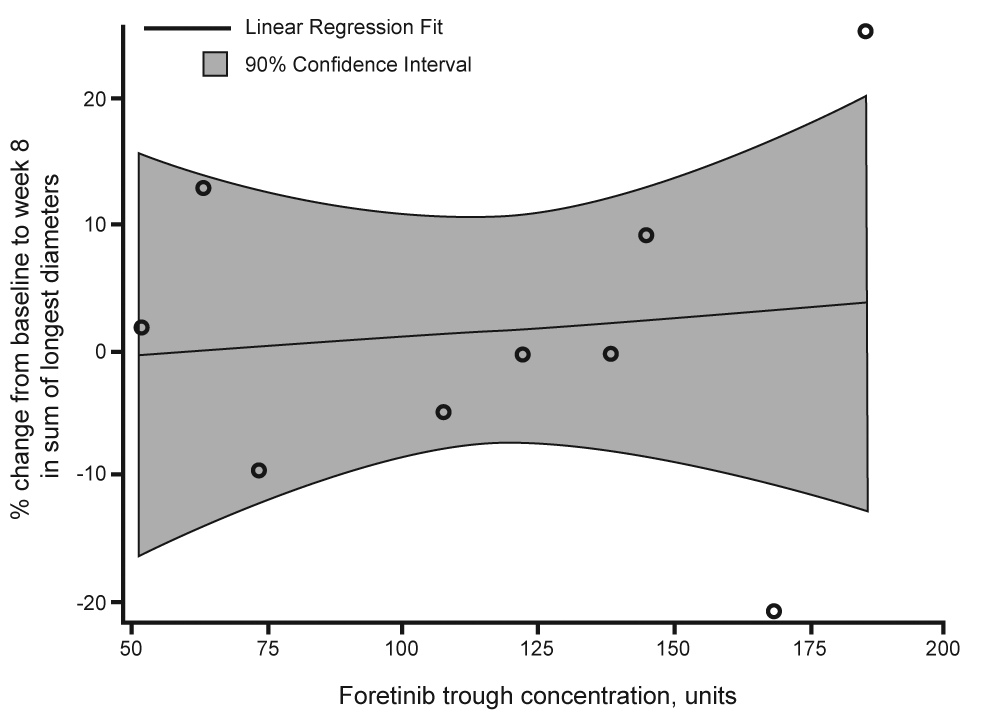

Supplement: Supplementary file 2 — (TIFF 756 kb) [file 10637_2012_9861_MOESM1_ESM.tif]
